# The Internet Healthcare Coalition

**DOI:** 10.2196/jmir.2.1.e3

**Published:** 2000-03-05

**Authors:** John Mack

**Keywords:** Internet, Ethics, Quality of Health Care

## About the Internet Healthcare Coalition

As individuals take a larger role in managing their own healthcare, more consumers are independently seeking out health information via the Internet. Health professionals are also turning to the Internet to keep informed and interact with their patients. Given the vast amount of healthcare data available on the Internet, the Internet Healthcare Coalition was created to promote quality health resources online and to ensure that consumers and professionals are able to find reliable, quality information online.

The Coalition is an international, non-partisan, non-profit organization dedicated to promoting quality healthcare resources on the Internet. Founded in 1997, the Coalition's membership represents every sector of the Internet health space, including consumers, patient advocates, commercial developers of health information, health professionals, medical librarians, government officials, and pharmaceutical manufacturers. The goal of the Coalition is to educate healthcare consumers, health professionals, and others about the evolving issues relating to the quality of Internet health resources and information.

The Coalition is dedicated to:

- Educating healthcare consumers, professionals, educators, marketers, and both healthcare and mainstream media, as well as public policymakers on the full range of uses of the Internet - current and potential - to deliver high-quality healthcare information and services.
- Furnishing clear models, not only of good and bad sources of online healthcare information and services, but of the potentially disparate methods of evaluating disparate sources of information - from product- or disease-information sites developed by regulated manufacturers, to peer-reviewed electronic publications and patient support groups.
- Publicizing and promoting the use of currently available resources and developing new resources that exemplify ethical, innovative, and high-quality uses of the Internet to deliver healthcare information and services.
- Acting as a representative of our constituencies in areas of mutual concern before public policymakers and with the media.

The Coalition consults with various government agencies including the World Health Organization (WHO), the US Food and Drug Administration (FDA), and the US Federal Trade Commission (FTC) on broader-based efforts to promote credible healthcare information and combat health fraud online. To that end the Coalition formed an Internet Health Fraud Resources Working Group which will work with the FTC to develop, coordinate, and promote a global online health-fraud reporting web site, which will link to authorities with jurisdiction over illegal or fraudulent online healthcare activities. Using this resource, consumers and healthcare professionals anywhere in the world can be sure that their complaints are transmitted to the appropriate authorities for action.

The Coalition holds an annual conference, "Quality Healthcare Information on the Net," each year in October or November for key thought leaders in the healthcare industry and government representatives to exchange their thoughts and opinions about the problems associated with and solutions to healthcare and the Internet. In October 1999, responding to calls from within the Internet Health community, the Coalition launched its ongoing "eHealth Ethics Initiative" to provide a forum for the development of a universal set of ethical principles for health-related Web sites.

The Coalition can be found at http://www.ihealthcoalition.org

